# Medical and Philosophical News

**Published:** 1781-02

**Authors:** 


The Memoirs of the Academy of Sciences at Berlin, for 1779, lately publiſhed, contain ſome account of the late Dr. J. H. Pott; this celebrated Chemiſt was born at Halberſtadt in 1692, and graduated at Halle in Saxony. His inaugural diſcourſe was on the ſulphur of metals. In 1718 he was admitted of the Academy, and about the ſame time was appointed Profeſſor of the Theory of Chemiſtry in the College of Phyſic and Surgery at Berlin. He died on the 29th of March 1777, aged eighty-five years, leaving ſeveral daughters. His *Lithogeognoſia* and other writings are well-known to the chemical reader.

